# Investigation of a Novel Multicomponent Mycotoxin Detoxifying Agent in Amelioration of Mycotoxicosis Induced by Aflatoxin-B1 and Ochratoxin A in Broiler Chicks

**DOI:** 10.3390/toxins13060367

**Published:** 2021-05-21

**Authors:** Vasilios Tsiouris, Panagiotis Tassis, Jog Raj, Tilemachos Mantzios, Konstantinos Kiskinis, Marko Vasiljević, Nikola Delić, Evanthia Petridou, Georgia D. Brellou, Zoe Polizopoulou, Nikolaos Mittas, Ioanna Georgopoulou

**Affiliations:** 1 Unit of Avian Medicine, School of Veterinary Medicine, Aristotle University of Thessaloniki, 54627 Thessaloniki, Greece; mantzios@vet.auth.gr (T.M.); kiskinik@vet.auth.gr (K.K.); ioannag@vet.auth.gr (I.G.); 2 Clinic of Farm Animals, School of Veterinary Medicine, Aristotle University of Thessaloniki, 54627 Thessaloniki, Greece; ptassis@vet.auth.gr; 3 Patent Co, DOO., Vlade Cetkovica IA, 24211 Misicevo, Serbia; jog.raj@patent-co.com (J.R.); marko.vasiljevic@patent-co.com (M.V.); 4 Institute for Animal Husbandry, Autoput 16, P. Box 23, 11080 Belgrade-Zemun, Serbia; delicnikola68@yahoo.com; 5 Laboratory of Microbiology and Infectious Diseases, School of Veterinary Medicine, Aristotle University of Thessaloniki, 54124 Thessaloniki, Greece; epetri@vet.auth.gr; 6 Laboratory of Pathology, School of Veterinary Medicine, Aristotle University of Thessaloniki, 54627 Thessaloniki, Greece; mprellou@vet.auth.gr; 7 Diagnostic Laboratory, School of Veterinary Medicine, Aristotle University of Thessaloniki, 54627 Thessaloniki, Greece; poliz@vet.auth.gr; 8 Department of Chemistry, School of Science, International Hellenic University, 65404 Kavala, Greece; nmittas@chem.ihu.gr

**Keywords:** aflatoxin, ochratoxin, clinoptilolite, silymarin, *Bacillus subtilis*, *Bacillus licheniformis*, *Saccharomyces cerevisiae*, broiler chickens, gut health

## Abstract

The present study was designed to determine the efficacy of a novel multicomponent mycotoxin detoxifying agent (MMDA) containing modified zeolite (Clinoptilolite), *Bacillus subtilis*, *B. licheniformis*, *Saccharomyces cerevisiae* cell walls and silymarin against the deleterious effects of Aflatoxin B_1_ (AFB_1_) and Ochratoxin A (OTA) in broiler chicks. A total of 160 one-day-old Ross 308^®^ broiler chicks were randomly allocated in four treatment groups, with four replicates, according to the following experimental design for 42 days. Group A received a basal diet; Group B received a basal diet contaminated with AFB_1_ and OTA at 0.1 mg/kg and 1 mg/kg, respectively; Group C received a basal diet contaminated with AFB_1_ and OTA and MMDA at 1 g/kg feed, and Group D received a basal diet contaminated with AFB_1_ and OTA and MMDA at 3 g/kg feed. Results showed that ingested mycotoxins led to significant (*p* ≤ 0.05) reduction in body weight and feed conversion from 25 days of age, induced histopathological changes, increased the pH of the intestinal content, and altered the biochemical profile of birds with significantly (*p* ≤ 0.05) increased aspartate aminotransferase (AST) values (*p* ≤ 0.05). On the other hand, the supplementation of MMDA significantly (*p* ≤ 0.05) improved the feed conversion ratio (FCR) during the second part of the study, diminished biochemical alterations, reduced pH in jejunal and ileal content, and *E. coli* counts in the caeca of birds (*p* ≤ 0.05). It may be concluded that the dietary supplementation of the MMDA partially ameliorated the adverse effects of AFB_1_ and OTA in broilers and could be an efficient tool in a mycotoxin control program.

## 1. Introduction

Mycotoxicosis was first described in 1960 when a shipment of Brazilian peanut meal was used in the diets of poultry and other domestic animals in the UK. This caused an outbreak of an unknown disease that killed more than 100,000 turkeys and was reported as the Turkey “X” Disease. Later, mycotoxins, which are a group of fungal secondary metabolites with diverse biological activity, were identified as the etiological factor [1]. The term “mycotoxin” derives from the Greek words “mykes”, which means “fungus” and “toxini”, which means “toxin”. Mycotoxins, depending on their chemical structure are capable of inducing adverse effects on human and animal health [2]. These low-molecular weight substances could enter the food chain, via contaminated food or feed, when raw materials like corn, wheat, peanuts, and sorghum are infected by toxigenic fungi in field, harvest, storage, or transport conditions [3]. In addition, mycotoxin residues in livestock products, such as meat, milk, and eggs, may lead to deleterious effects on consumer health. Thus, the mycotoxins menace has a worldwide impact on human and animal health, whereas raw materials’ contamination by mycotoxins, and results in a significant economic burden to agriculture [4].

Among almost 500 identified mycotoxins, Aflatoxins (AFs) are considered the most hazardous for human and animal health. AFs are mainly produced by fungi belonging to the genus *Aspergillus* spp., particularly *A. flavus*, *A. parasiticus* and *A. nomius*, which primarily grow in regions with tropical or subtropical climate [5]. From nearly 20 chemical substances that belong to this group, Aflatoxin B_1_ (AFB_1_) consists of the most detected mycotoxins in cereals and has the greatest toxicity. In both, humans and animals, AFB_1_ is reported to cause carcinogenic, mutagenic or teratogenic effects, and is therefore categorized as human carcinogen group I [6]. In a variety of poultry species, such as chickens, turkeys, ducks and quails, certain exposure to high dosages of AFB_1_ may induce clinical signs, like mortality, decreased egg production, reduced body weight, and feed efficacy [7]. However, in practice, AFB_1_ is usually detected at low concentrations in poultry feeds, which may lead to subclinical effects, including slightly reduced production, immunosuppression, and vaccination failure [8].

Ochratoxins are secondary metabolites, produced by fungi mainly belonging to genera *Aspergillus* spp. and *Penicillium* spp. The group consisted of three metabolite forms (A–C), with Ochratoxin A (OTA) being the most important. OTA is classified by the International Agency for Research on Cancer as a possible human carcinogen (Group 2B) and is associated with immunotoxic, neurotoxic, hepatotoxic and embryotoxic effects in both humans and animals. OTA is a very stable substance in high thermal conditions and thus is very difficult to eliminate by physical methods, once occurred in the feed [9]. OTA is primarily produced under storage conditions and frequently co-occurs with other mycotoxins [10].

Long-term epidemiological surveys indicate that a large fraction of global feedstuffs is exposed to more than one mycotoxin [11,12]. The co-occurrence of mycotoxins may lead to antagonistic, additive, or synergic effects. In broilers, AFB_1_ is the most potent hepatotoxic associated with an enlarged, pale and friable liver, with histological findings of fatty infiltration and bile duct proliferation [13]. On the other hand, OTA is principally nephrotoxic and is associated with kidney enlargement and hyperplasia of the tubular epithelium [14]. Both mycotoxins have been further associated with significant alterations in the biochemical profile of birds, decreased nutrient digestibility, suppression of digestive enzymes and decreased serum proteins—in sum, affecting productivity and profitability [3,8,9,15,16].

However, when AFs and OTA co-existed in the poultry feeds, their interaction leads to less additive and more antagonistic effects in biochemical parameters of birds, including total proteins, albumins, cholesterol, creatinine, and uric acid [17,18,19]. Moreover, the combination of AFB_1_ and OTA can lead to less apparent hepatic and more severe kidney lesions, which may be a burden in achieving diagnosis [20]. This can be attributed to the fact that AFB_1_ and OTA show weak cytotoxic effect in liver cell lines, when are in combination than alone, suggesting an antagonistic interaction [21]. In contrast, in most of the studies, AFs and OTA induced synergistic and/or additive interactions in mortality, body weight, feed intake, egg production, embryo abnormalities, and atrophy of lymphoid organs [17,18]. A wide variation of the results may be attributed to the different experimental designs, which varied in the concentration of ingested mycotoxins and the time of exposure. So far, there are only a few studies focused on the long-term exposure of broilers to combinations of AFB_1_ and OTA in concentrations as low as naturally found in foodstuffs.

Recognition that mycotoxins affect health and productivity in poultry farming has set maximum levels or guidance values for mycotoxins. According to directive 2002/32/EC on undesirable substances in animal feed, the limit imposed for AFB_1_ in complete feed, with 12% moisture content, for poultry (with the exception of young ones) is 0.02 mg/kg [22]. Furthermore, according to directive 2006/576/EC, guidance values for complementary and complete feedstuffs for poultry are 0.1 mg/kg OTA [23]. The Food and Drug Administration (FDA) has also set maximum levels or guidance values that vary according to the animal species, mycotoxin, raw material, etc., and may differ from EU regulations [5,19].

Despite many years of research and interventions in pre-harvest, harvest and post-harvest levels, mycotoxins continue to be a considerable threat to the poultry industry [7]. As harvest practices and storage conditions remain improper, the pursuance of more practical and effective methods to detoxify mycotoxin-contaminated feed and foodstuffs at a farm level become increasingly significant to ameliorate their public and animal health risk issues. There is no single method for the deactivation of mycotoxins in feed as a result of their great variety. Therefore, a holistic approach is needed to identify the risk and adopt the best strategy, without compromising the feed quality.

One of the approaches to this global concern is “binding” of mycotoxins in the gastrointestinal tract of animals and reducing their bioavailability, with the use of inorganic adsorbents, such as clay minerals, bentonite and zeolite in animal feed [24]. Clinoptilolite is a natural zeolite with unique and outstanding physical and chemical properties. Due to its diverse structure, with cavities and canals, clinoptilolite has a large surface for water absorption and cation enhancing. Specifically, clinoptilolite has remarkable adsorption indexes in vitro, with more than 80% for AFB_1_ [25,26]. However, even though adsorbents like clinoptilolite effectively bind mycotoxins as AFB_1_, they are not equally efficient for other mycotoxins, such as trichothecenes. Therefore, alternative approaches for the amelioration of mycotoxicosis adverse effects in broiler chicks are required [8]. Novel approaches also include the creation of modified forms (e.g., modification of surface properties) of such substances with the aim of increased adsorbing ability. A previous in vitro study suggested that organically modified natural zeolite showed greater adsorbing ability against OTA and Zearalenone (ZEN) in comparison to the unmodified form [27].

Moreover, the use of specific microorganisms, including bacteria, fungi and yeast, or enzyme systems that effectively bio-transform non-binding mycotoxins into some less-toxic metabolites, is a promising alternative. Rao et al. [28] reported that *Bacillus licheniformis* CFR1 shows an AFB_1_ degradation of more than 90% when tested in vitro, whereas yeast cell wall containing beta-glucans and mannan oligosaccharides can efficiently bind with AFB_1_ up to 90% [29].

However, only limited research studies exist, using a combination of the previously mentioned approaches that investigate their ameliorating effects against AFB_1_ and OTA when co-occurring in broilers feed at realistic or occasional levels. If the dietary supplementation of a novel multicomponent mycotoxin detoxifying agent could ameliorate the negative effects induced by Aflatoxin-B1 and Ochratoxin A on the intestinal ecosystem, then the performance of broiler chicks could be improved. Thus, this study aims to investigate the efficacy of a novel multicomponent mycotoxin detoxifying agent (MMDA) containing modified zeolite (clinoptilolite), *B. subtilis*, *B. licheniformis*, *S. cerevisiae* cell wall and silymarin, on the physicochemical properties of the intestinal content, gut microbiota, histology, serum biochemistry and performance in broiler chickens ingesting AFB_1_-and OTA-contaminated feed.

## 2. Results

### 2.1. Performance

Daily clinical examination of birds revealed sporadic clinical signs, indicative of mycotoxicosis, including ruffled feathers, diarrhea and growth retardation in groups that received contaminated feed. Clinical signs started on day 17 and were continued until the end of experiment (42nd day). During the experimental period, no mortality was recorded among the tested groups. The effect of mycotoxins and multicomponent mycotoxin detoxifying agent (MMDA) on the performance of birds per treatment group is shown in Table 1.

Feed contamination with Aflatoxin B1 (AFB1) and Ochratoxin A (OTA) did not significantly affect the body weight (BW) of birds for the first two weeks when compared to the controls. However, from the 25th day of the trial and onwards, the BW of animals receiving mycotoxins either with or without the test product was significantly lower than the control group (*p* < 0.001 for all comparisons). On the other hand, average daily weight gain (ADWG) mean values did not show significant differences among groups at any point of the study. Feed conversion ratio (FCR) alterations between groups and time periods showed a quite different pattern. At the mid-point of the study (25 days of age), the FCR of the three groups receiving AFB_1_ and OTA were significantly reduced when compared to group A (*p* = 0.003 group B vs. A; *p* = 0.001 group C vs. A; *p* = 0.002 group D vs. A). On the contrary, at the end time point of the study (42 days of age) the FCR improved in the groups receiving the test product (C and D group) in comparison with the B group. The comparisons among groups for the total trial period suggested a numerical improvement of FCR and Adjusted FCR in C and D groups in comparison with the B group. Nevertheless, the FCR comparisons showed a trend for statistically significant improvement in C and D groups when compared with the B group (*p* = 0.076 and *p* = 0.01, respectively).

### 2.2. Serum Biochemical Analysis

The effect of ingested mycotoxins and treatments on the serological profile of broilers at 25th and 42nd day of age is displayed in Table 2.

Total protein, albumin, alkaline phosphatase (ALP), alanine aminotransferase (ALT) and gamma-glutamyl transferase (γ-GT) values in all experimental groups were not significantly different neither at 25, nor at 42 days of age. A significant increase of glucose levels was observed in group D that received the greater dosage level of the test product in feed (3 g test product/kg feed) when compared with group B (*p* = 0.023). Such observation was not evident for the lower dosage level group C (1g test product/kg feed). On the other hand, ALT values were significantly improved in group D, irrespective of the factor time, when compared with group B (*p* = 0.007). Both of the abovementioned evidence sources support a liver-protective effect of the test product when added at the greatest concentration level. However, aspartate aminotransferase (AST) values were significantly increased in the groups that received mycotoxins (B, C and D) in comparison with group A at both sampling time points (*p* < 0.001) for all comparisons except comparison between groups D and A at first sampling: *p* = 0.007).

### 2.3. Gross Lesion Score

The results of the gross lesion score are presented in Table 3 and revealed insignificant differences in intestinal and liver lesions among experimental groups. However, gizzard lesions were significantly lower (*p* = 0.042) in the group that received MMDA at 3 g/kg feed when compared to other experimental groups.

### 2.4. Physicochemical Measurements of Intestinal Content

The results of the pH and viscosity values of the intestinal content are shown in Table 3. The ingestion of AFB_1_ and OTA led to a significant increase in the pH value of the duodenal and jejunal content in comparison with the control group. However, the addition of MMDA at 3 g/kg feed significantly ameliorated the increase of the pH value in the jejunum (*p* = 0.001). The pH of the ileal content was significantly lower in groups that received MMDA either at high or low dosage level, when compared to the control group (*p* < 0.001 and *p* = 0.001, respectively). Moreover, the addition of MMDA in 3 g/kg led to a significant increase in the caecal pH. The viscosity of jejunal and ileal content was insignificantly different among groups. However, the highest jejunal and ileal viscosity values were noted in groups that ingested mycotoxins without the test product, whereas the lowest numerical values were observed in group C.

### 2.5. Histopathological Findings

**Figure 1:**
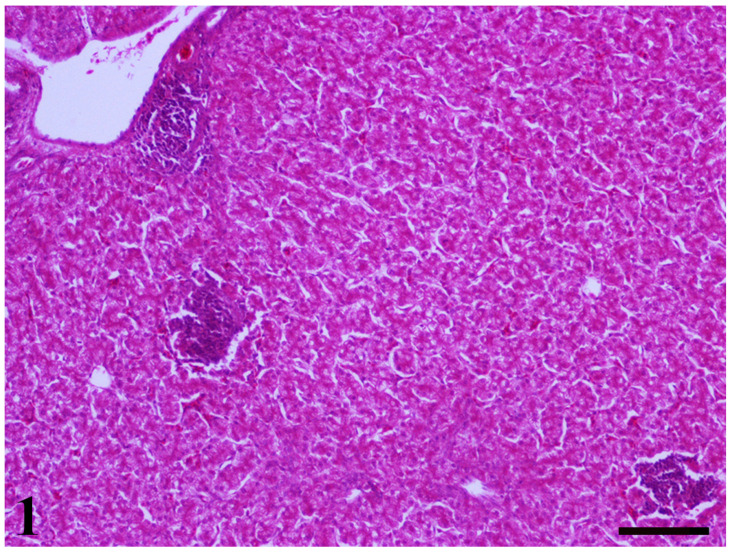
A liver section of 25-day-old broiler chicks dietary supplemented with Aflatoxin—B_1_ (AFB_1_) and Ochratoxin A (OTA). Three foci with lymphocytic-plasmacytic inflammation, as well as the presence of diffuse vacuolar degeneration of hepatocytes are obvious (H–E, Bar = 100 μm).

Samples from the liver, kidney, and intestine, based on specific gross pathology criteria, were collected and pooled from animals of each group for further histopathological analysis. All liver samples from all groups showed multifocal inflammation and hepatocellular vacuolation on day 25 (Figure 1), whereas the multifocal inflammation was the predominant finding in livers of all groups on day 42. In the groups that received MMDA, bile duct hyperplasia was noted in both samplings, whereas the most severe was present in the group that received the lowest dose at the first sampling. At 42 days of age, bile duct hyperplasia was noted in controls and group B, whereas the lesions were presented focally in Group C. Moreover, myelocytes were presented in the liver of all groups, whereas fatty degeneration and fibrosis alongside hemorrhages were noted in Groups D and C, respectively. Taken together, the liver of the group that received MMDA at 1 g/kg feed showed more obvious signs of mycotoxins-attributed negative effects on the 25th day of age when compared with the others.

**Figure 2:**
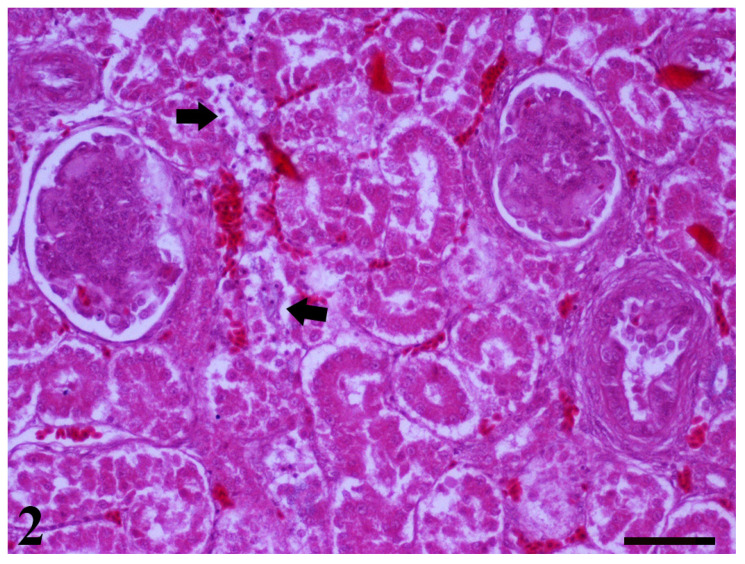
A kidney section of 25-day-old broiler chicks dietary supplemented with AFB1 and OTA, is pictured. Two glomeruli present glomerulonephritis. In addition, areas with necrosis of tubular epithelium are also observed (arrows) (H–E. Bar = 50 μm).

On day 25, kidney samples of all groups showed glomerulonephritis alongside focal necrosis (Figure 2). Moreover, in the kidneys of Group B and D, vacuolar degeneration findings were also noted. At 42 days, kidneys from the control group revealed an absence of any histopathological findings, whereas samples from groups B and C showed focal necrosis and vacuolar degeneration as predominant findings. The only histopathological finding noted in group D was enlarged epithelial cells. Taken together, histological findings in the kidneys on the 25th day of the study did not present any significant differences among groups. On day 42, the histological picture in the kidneys of birds that received MMDA at 3 g/kg seemed to be somewhat improved in terms of pathological findings in comparison with group B.

The crypt hyperplasia finding was present in small intestine samples of groups that received contaminated feed, but the overall picture of samples from groups that received the test product (i.e., C and D groups) was milder, especially on the 42nd day of the study. Mild lymphocytic-plasmacytic inflammation was present also in groups A and B. Focal lymphocytic-plasmacytic inflammation with a mild occurrence of macrophages and surface epithelial necrosis as well as focal atrophy and necrosis of villi, was observed in group C. Focal atrophy of the villi without findings of necrosis was also present in group D samples. Increased goblet cells were present in group B samples and myelocytes in group D samples.

### 2.6. Microbiological Analysis

Microbiological analysis of caecal content at the end of the study period is presented in Table 4. The counts of *E. coli* were not significantly affected by the ingestion of AFB_1_ and OTA in group B when compared with controls. However, the addition of MMDA at the concentration of 1 or 3 g/kg of feed, significantly reduced the *E. coli* counts in the caecal content of birds when compared with group B (*p* = 0.006 group C vs. B; *p* = 0.012 group D vs. B). Counts of *Clostridium* spp., *Lactobacillus* spp. and *Bifidobacterium* spp. were not significantly affected by the ingestion of mycotoxins or the test product.

## 3. Discussion

Mycotoxins are considered the most dangerous contaminants of animal’s feeds as even low levels could lead to deleterious effects. As a mycotoxin occurs in feed it is very difficult to eliminate [30]. Among different approaches, the use of biological detoxification at the household level is getting attendance. The present study aimed to document the efficacy of a novel multicomponent mycotoxin detoxifying agent (MMDA) containing modified zeolite (Clinoptilolite), *B. subtilis*, *B. licheniformis*, *S. cerevisiae* cell wall and silymarin fed to broiler chickens ingesting contaminated feed with Aflatoxin B1 (AFB1) and Ochratoxin A (OTA). Results suggested a partial improvement of performance and biochemical parameters, as well as reduced *E. coli* counts in groups treated with the test product when compared with the group that received only AFB_1_ and OTA. The group that received the test product at greater dosage (group D) showed an overall more noticeable improvement of particular parameters in comparison with the low-dose group (group C).

### 3.1. Performance

Several studies have been conducted to determine the adverse synergistic effect of AFB_1_ and OTA on broiler performance [15]. There is a general agreement that ingestion of the abovementioned mycotoxins, alone or in combination reduce body weight, feed intake and increase feed conversion ratio. Elaroussi et al. [31] reported a significant reduction of body weight and feed conversion in broilers fed 0.4 mg/kg or 0.8 mg/kg OTA. Furthermore, Zhao et al. [32] recorded a significant reduction of weight gain (10%) at 21 days of exposure in broilers ingested by 1 mg/kg AFB_1_ in their diets. Thus, it is not surprising that, in the present study, the dietary supplementation with 0.1 mg/kg AFB_1_ and 1 mg/kg OTA in broiler diets significantly compromised the BW of birds from 25 days of exposure. Moreover, the ingestion of AFB_1_ and OTA, led to a significant increase in the feed conversion ratio (FCR). The mechanism behind the adverse effects of ingested mycotoxins in BW of birds has not been fully elucidated; however, the aforementioned mycotoxins have been associated with anorexia, listlessness, and metabolic changes, as a result of the liver damage and the decrease of the activity of digestive enzymes in combination with the decrease of nutrient digestibility due to the changes on the intestinal environment [33,34]. In addition, AFB_1_ inhibits the digestibility of fats by reducing both enzyme activity and bile acid production necessary for their digestion and absorption [35].

The addition of MMDA product, ameliorated the adverse effect of AFB_1_ and OTA on the FCR. The ability of clinoptilolites to diminish the adverse effects of mycotoxins in broiler performance has been previously described. Orgus et al. [36] reported that the addition of clinoptilolite at levels of 15 g/kg feed significantly ameliorated the deleterious effects of 2.5 mg/kg AFB_1_ on feed consumption and body weight gain. Moreover, Nedeljković-Trailović et al. [37] saw that the addition of the modified clinoptilolite at a concentration of 0.2%, was possible to ameliorate the negative effects of a contaminated diet with 2 mg/kg OTA in broilers. In addition, the microbial ingredients of MMDA product, *B. subtilis* and *B. licheniformis*, could degrade the mycotoxins and reduce their absorption, whereas the *Saccharomyces cerevisiae* cell wall had shown sorption capability against OTA [38,39]. Furthermore, it has been suggested that treatment with silymarin can be effective in counteracting the negative effects of AFB_1_ intoxication on the performance of broiler chicks [40]. Finally, it is noteworthy that most of the studies have been performed with greater concentrations of AFB_1_ or OTA; thus the effects on performance of broiler chicks were more pronounced, and the respective improvements provided by adsorbents were more noticeable.

### 3.2. Biochemical Investigation

Studies conducted during the last 15 years revealed that AFB_1_ and OTA are associated with significant alterations in the biochemical profile of broilers. Mycotoxicosis can be suspected by determining changes in serum biochemistry parameters before clinical symptoms become apparent. The change of these serum biochemistry parameters is indicative of liver pathology [41]. Denli et al. [42] reported that ingestion of 1 mg/kg AFB_1_ in broiler feed led to decreased total protein and increased alkaline phosphatase (ALP) values in the serum of birds. Moreover, decreased serum albumin, cholesterol levels and increased activity of hepatic enzymes, including ALP, alanine aminotransferase (ALT), gamma-glutamyl transferase (γ-GT), have been noted when AFB_1_ added in broiler diets in concentrations up to 1 mg/kg [15]. In our study, the addition of AFB_1_ and OTA in concentrations of 0.1 and 1 mg/kg, respectively, did not significantly alter the biochemical profile of broilers either on 25 days or at 42 days of life. However, a significant increase in aspartate aminotransferase (AST) values, as compared with the control group was noted in both samplings.

Our results are in agreement with Pappas et al. [19] who did not observe significant differences in total serum proteins, albumin, cholesterol, ALP and γ-GT in broilers ingested by a combination of 0.1 mg/kg AFB_1_ and OTA. The liver is the main target organ involved in the detoxification of mycotoxins alongside the kidneys. Blood serum enzymes, including ALP, ALT, AST and γ-GT, are important blood biomarkers of liver activity [43]. These enzymes mainly occurred in the cytoplasm and mitochondria of liver cells [44]. Their elevation in blood serum may be due to disruption of hepatic cells as a result of necrosis or a consequence of altered membrane permeability, caused by the ingestion of mycotoxins [45]. In addition, the increase of the levels of serum enzymes might be expounded because of hepatocyte degeneration and subsequent leakage of enzymes into the bloodstream, as well as biliary cholestasis and the hyperplasia of bile ducts [41]. The addition of MMDA at the concentration of 3 g/kg feed resulted in a significant reduction of AST only at the first blood sampling and ALT values without treatment × time interaction in group D when compared with group B, whilst glucose values of group D were increased in comparison with group B without treatment × time interaction. Silymarin extract has been reported as a hepatoprotective agent in poultry and was able to reduce the liver effects of AFB_1_ in broilers [39]. In addition, silymarin is known to be a potent antioxidant and it can inhibit the cytochrome P450 system, and consequently inhibit AFB_1_ activation [40]. Moreover, *Saccharomyces cerevisiae* cell wall glucans, can act as nutritional aids and growth promotors, and bind mycotoxins and could act synergically with the modified zeolite adsorbing ability [39].

### 3.3. pH and Viscosity

The main factors that determine the pH of intestinal digesta are the feed composition, the gastrointestinal secretions and the volatile fatty acids, produced by intestinal microbiota. Thus, any factor altering the intestinal mucosa and microbiota could influence the pH of intestinal digesta. The manipulation of digesta pH in broiler chickens could act as a tool to manage the potential for optimum gut health and maximum nutrient absorption since the reduction of the pH may inhibit the growth of acid-sensitive bacteria, such as *Salmonella* spp., *Campylobacter* spp. and *Clostridium perfringens* [46]. In our study, the addition of AFB_1_ and OTA induced a significant increase in the pH of the duodenal and jejunal content. However, the addition of MMDA at both dosage levels significantly reduced the pH value of jejunal and ileal content. Our results are in agreement with Ismail et al. [47], who reported significantly lower pH values in the ileum content of the group received zeolite than those in the control group. This could be explained by the ability of zeolites to attract and buffer excess protons that cause acidity. In addition, the probiotic bacteria of MMDA product (*B. subtilis* and *B. Licheniformis*) are able to metabolize soluble nondigestible carbohydrates into short-chain fatty acids (SCFAs) and lactate which consequently lowers the pH, while the prebiotic *Saccharomyces cerevisiae* cell wall promotes the proliferation of lactic acid bacteria in the intestine contributing also to the reduction of pH [1,46].

Viscosity, a physicochemical property of the intestinal digesta, is associated with the feed materials, mainly the undigested part and the digestive chyme. Increased viscosity in the gastrointestinal tract of poultry has been shown to negatively interfere with digestion and absorption of nutrients and consequently reduce performance [46]. In particular, it is assumed that increased intestinal viscosity depresses nutrient digestibility by interference with the diffusion of digestive enzymes to their substrates and with the movement of digesta across the intestinal lumen. In addition, a higher intestinal viscosity increases the average retention time of the digesta, which is likely to create a favorable environment for bacterial activity, because the flow of digesta is reduced and the amount of undigested material in the intestinal tract is increased. This gives more time to the microbes to colonize the small intestine and results in greater competition with the host for nutrients. It has been reported that clinoptilolites, due to their diverse structure with cavities and canals, increase absorption surface for water and osmotically active cations, which may lead to an increase in the intestinal viscosity depressing nutrient digestibility [48,49]. However, in our study the addition of MMDA, based on modified clinoptilolite did not significantly alter the viscosity of intestinal content in jejunum and ileum.

### 3.4. Gut Microbiota

The gut microbiota plays a vital role in maintaining health and influences the overall performance of chickens. In addition, gut microbiota limits and controls the colonization of foodborne pathogens by a competitive exclusion process. It acts through diverse mechanisms, such as providing nutrients, preventing pathogen adhesion to host cells, interacting with host immune systems, affecting the gut morphological structure, and producing organic acids and antimicrobial agents [50,51]. The mycotoxins have profound interactions with the gut microbiota, particularly in animals, since some mycotoxins exhibit antimicrobial properties, while others are biotransformed by microbiota [52]. It is well known that the absorption rate of OTA is low in poultry, and the unabsorbed OTA could reach the hindgut to interact with the intestinal microbiota [9]. Ouethrani et al. [53] noted that OTA caused a specific, but lasting, loss of the beneficial bacteria in the human colon, including *Lactobacillus* spp. and *Bifidobacteria*, which may lead to a less competitive environment for the growth of pathogenetic bacteria like *E. coli* and *Salmonella*. In our study, the ingestion of AFB_1_ and OTA increased the counts of *E. coli* in the ceca of birds; however *Lactobacillus* spp. and *Bifidobacteria* counts were not significantly affected, and this hypothesis cannot be supported. Our results are in line with Jahanian et al. [54], who noted that ileal populations of *E. coli*, *Salmonella* spp., *Klebsiella* spp. and total negative bacteria were markedly elevated by increasing the aflatoxin level in broilers feed. These high numbers of *E. coli* could be attributed to the favorable intestinal environment created by the increase of the intestinal pH.

Also, clinoptilolites can absorb certain microorganisms and their toxins. In many in vitro and in vivo studies, the sorption capacity of clinoptilolites for pathogenetic bacteria, including *E. coli* and *Salmonella* has been highlighted [55,56]. Modification of clinoptilolites with organic acids, has been reported to increase the bactericidal effect against *E. coli* and *Salmonella* spp., as a result of the increased hydrophobicity of the mineral surface [57]. In our study, the dietary supplementation of the novel multicomponent mycotoxin detoxifying agent “MMDA”, containing modified zeolite (Clinoptilolite), *B. subtilis*, *B. licheniformis*, *Saccharomyces cerevisiae* cell wall and silymarin, ameliorated the increase of *E. coli*, which agrees with the previously mentioned studies.

## 4. Conclusions

Based on the results of the study, we could suggest that the addition of the test product in feed moderately ameliorated the deleterious effects of AFB_1_ and OTA at concentrations of 0.1 mg/kg feed and 1 mg/kg feed, respectively, in broiler chicks. In particular, the added mycotoxins deteriorated the FCR, as a result of poor nutrient digestion and absorption, whereas the use of the test product limited the increase of FCR that mycotoxins caused and showed a mild protective effect against mycotoxicosis, in particular in liver function. In addition, the use of MMDA reduced the *E. coli* counts in the caeca as well as limited the increase of the pH value of jejunal and ileal content, which could act as a favor environment for gut pathogens, such as *E. coli*, *C. perfringens*, *Salmonella* spp. and *Campylobacter* spp. It can be concluded that the MMDA product, a novel multicomponent myco-toxin detoxifying agent, could positively affect the intestinal ecosystem and thus could be an important part of a mycotoxin control program.

## 5. Materials and Methods

### 5.1. Animals and Ethics

The experimental study was performed in the experimental facilities of the Unit of Avian Medicine, School of Veterinary Medicine, Aristotle University of Thessaloniki (AUTh), Greece. Husbandry, euthanasia, experimental procedures, and biosecurity precautions were conducted in accordance with Council Directive (2010/63/EU) [58] and the Greek legislation governing experimental animals and were approved by the Ethical Committee of AUTh (31856(108)).

### 5.2. Test Product

The test product was a novel multicomponent mycotoxin detoxifying agent (MMDA; Patent Co DOO, Misicevo, Serbia) containing modified zeolite (Clinoptilolite), *B. subtilis*, *B. licheniformis, S. cerevisiae* cell wall and silymarin.

### 5.3. Mycotoxin Production

Corn spiked with 12.3 mg/kg AFB_1_ and 188 mg/kg Ochratoxin A (OTA) was provided by the sponsor of the study (Patent Co, Misicevo, Serbia) and used for the contamination of basal feed by partial replacement of non-contaminated corn. Contaminated corn was produced after incubation with *Aspergillus ochraceus* and *Aspergillus parasiticus* at 25 °C with 13% moisture content for 15 days. After incubation, the corn was autoclaved, dried, and grounded to powder form. The amount of Aflatoxin-B1 (AFB_1_) and OTA in spiked corn was quantified using liquid chromatography–tandem mass spectrometry (LC–MS/MS).

### 5.4. Feed Preparation and Analysis

Contaminated feed was prepared with the inclusion of contaminated raw material in flour form into the two types of feed that were used during the trial. The final target was contamination levels of 0.1 mg/kg feed for AFB_1_ and 1 mg/kg feed for OTA, which equals to 5 (AFB_1_) and 10 (OTA) times greater levels than the EU suggested maximum levels [22,23]. Multi-mycotoxin analysis of aflatoxins (AFB_1_, AFB_2_, AFG_1_, AFG_2_), diacetoxyscirpenol (DAS), deoxynivalenol (DON), zearalenone (ZEN), OTA, fumonisins (FB_1_, FB_2_) and trichothecenes (T_2_, HT_2_) for the trial feeds was performed at an accredited testing laboratory (Agrolab RDS, Sindos, Greece), whereas feed nutritional analysis was performed in the Laboratory of Nutrition (School of Veterinary Medicine, AUTh). Mycotoxin analysis of feed was performed by LC/MS–MS following the method of Li et al. [59] for fumonisins detection and Ren et al. [60] for the rest of the toxins, as described by Tassis et al. [61]. Detection limits were 0.5 μg/kg for AFB_1_, AFB_2_, AFG_1_, AFG_2_, 1 μg/kg for OTA, T-2, HT-2 and DAS, and 10 μg/kg for ZEN, DON, FB_1_, FB_2_. Feed analysis was performed with a diode array near-infrared spectroscopy instrument (DA7250, PerkinElmer, Waltham, MA, USA) and included Weende constituents as well as starch, total sugars, calcium, phosphorus. Feed and mycotoxin analysis results are presented in Tables S1 and S2. Subsamples of each experimental diet were collected for analysis according to EC Regulation 152/2009 [62].

The production procedure of trial feeds was performed by gentle mixing of the test particles in the feed and inclusion to the remaining feed. Due to the relative toxicity of AFB_1_ to humans, special precautions were taken during the preparation and serving of trial feed to the animals in order to minimize the inhalation of dust particles by persons involved in the trial. Therefore, efforts to minimize dust formation during feed preparations and handling, as well as the use of appropriate biosafety equipment (masks) were used by the relative personnel when in contact with contaminated feed. To avoid contamination with previous productions, the feed was manufactured in an appropriate rank order starting with the control diet and with a neutral meal mixing in between each of the diets. The rank of feed production order was group A feed at first followed by B, C and D group’s feed, respectively. Among each production, a quantity of control corn was used for the removal of leftovers in the mixing mill.

### 5.5. Experimental Design

One hundred and sixty (160) one-day-old Ross 308^®^ (Aviagen^®^) broiler chicks were randomly allocated into four treatment groups of ten chicks each, with four replicates per group. Broilers in all groups consumed a specially formulated two-phase feed. A starter feed (Supplementary Table S1) was provided up to the 25th day of age (A-phase feed), followed by a grower feed (Supplementary Table S2) up to the end of the study period on the 42nd day of age (B-phase feed). No antibiotic growth promoters, organic acids and phytobiotics were used. Feed and drinking water were offered to all birds ad libitum throughout the experiment. The treatment groups were: Group A, received basal diets without addition of the test product (MMDA). Group B received basal diets contaminated with AFB_1_ and OTA, respectively, reaching concentrations of 0.1 mg/kg AFB_1_ feed and 1 mg/kg OTA feed approximately, without the addition of the test product. Groups C and D received contaminated feed with 0.1 mg/kg feed AFB_1_ and 1 mg/kg feed OTA with the addition of MMDA at concentrations of 1 g and 3 g/kg feed, respectively. The selection of contamination levels used in this study was based on the objective to test MMDA efficacy under realistic (AFB_1_) or occasional (OTA) contamination (AFB_1_: <0.3 mg/kg; OTA: <2 mg/kg) conditions [8].

Birds in each group were placed in a pen with a deep litter of wood shavings, which were previously sterilized in an autoclave at 121 °C for 20 min (Cyclomotic control, EA605A, American Sterilizer Company, Mentor, OH, USA). Each group was kept in a specially designed experimental room (Unit of Avian Medicine, Faculty of Veterinary Medicine, School of Health Sciences, Aristotle University of Thessaloniki), where the temperature, the relative humidity and the lighting program was controlled, following the recommendations of the breeding company (Aviagen^®^). Temperature and humidity were monitored in each room at two locations at bird level using a temperature-humidity record system (HOBO UX100-003 Temperature/Relative Humidity data logger, Onset Computer Corporation, 470 MacArthur Blvd., Bourne, MA 02532, USA).

### 5.6. Performance

All experimental animals were monitored daily by an experienced veterinarian for adverse clinical signs (vomiting, diarrhea, dyspnea, loss of appetite, depression, etc.). A double-blinded scheme was implemented, thus personnel occupied in clinical evaluation of trial animals and the respective personnel that performed laboratory analysis were unaware of trial grouping and treatments. Bodyweight (BW) was measured on the 1st, 7th, 15th, 25th, 32nd and 42nd day of age. Average daily feed intake (ADFI), Average daily weight gain (ADWG: (Liveweight (end)—Liveweight (initial)/days on trial)) and Feed conversion ratio (FCR: ADFI/ADG) were recorded. ADWG and FCR were reported for the periods of the 1st–25th day, 25th–42nd day and 1st–42nd. An Adjusted FCR (Adj. FCR) was calculated based on a standard reference weight of 2500 g, according to the following equation: Adj.FCR = FCR − Y.Y. = (Average slaughter weight − 2500)/50/100. Thus, an effect on the FCR of about 2 points (±0.2) for each 100 g difference between 2500 g and actual slaughter weight was adjusted. For each dead bird of the trial, the date, the age, the live weight and the possible cause of death were recorded.

### 5.7. Serum Biochemical Analysis

Twenty-four birds per treatment group per sampling day were bled via the jugular vein. The blood collected in 10 mL tubes and centrifuged for 3 min at 3000 rpm in order to obtain the serum. Serum albumin, total protein, cholesterol, glucose, alkaline phosphatase (ALP), alanine aminotransferase (ALT), aspartate aminotransferase (AST), gamma-glutamyltransferase (γ-GT) were measured with an automated clinical chemistry analyzer (Vitalab^®^ flexor, Vital Scientific NV, Spankeren/Dieren, The Netherlands) in the Diagnostic Laboratory of the School of Veterinary Medicine, AUTh.

### 5.8. Gross Lesion Score

The intestine, the gizzard and the liver were collected, macroscopically examined, and scored for gross lesions [63,64,65]. In particular, the intestines were macroscopically examined and scored for enteritis lesions following a 0–3 scoring system. Intestines received a score of 0, in the case of the normal intestinal wall, a score of 1 if hyperemia of intestinal wall and/or undigested intestinal content in the last third of intestine were present, a score of 2 if congestion and thickness of intestinal wall and/or watery intestinal content were present, and a score of 3 if hemorrhages of intestinal wall were noted [63]. Birds with intestinal lesions score greater than 1 were classified as enteritis positive. The gizzards were macroscopically examined and scored as described by Novoa-Garrido et al. [64] according to a zero to two scoring system as follows: 0 = healthy gizzard, if lesions were absent; 1 = moderately affected gizzard, if erosions in the koilin layer were present, and 2 = severely affected gizzard if ulcers of the cuticula of the gizzard were noted. The livers were macroscopically examined and scored for gross lesions using a 0–2 scale, as described by Tsiouris et al. [65], giving a score of 0, when no gross lesions were observed, a score of 1, when liver congestion and/or gallbladder distension and wall thickening and/or bile discoloration were observed, and a score of 2, when necrotic lesions in the liver were noted.

### 5.9. Physicochemical Measurements of Intestinal Content

The digesta of the duodenum, jejunum, ileum, and caecum from each bird were immediately collected after euthanasia in separate tubes and vortexed to obtain a homogenous content from each anatomical part of the intestine per bird. The pH of the duodenum, jejunum, ileum, and caecum from each bird was measured using a digital pH-meter (pH 315i, WTW Wissenschaftlich-Technische Werkstätten, Weilheim, Germany). Two readings were taken from each sample and the average was presented.

The viscosity of intestinal digesta was determined as described by Tsiouris et al. [66]. The homogenous content of the jejunum and the ileum from each bird was filled in separate Eppendorf tubes (1.5 mL). The tubes were centrifuged at 3000× *g* for 15 min to separate the feed particles from the liquid phase. Supernatants (0.5 mL) from each tube were taken and the viscosity was measured in a Brookfield DV-II+ PRO Digital Viscometer (Brookfield Engineering Laboratories, Stoughton, MA, USA). Two readings were taken from each sample and the average was expressed in centipoise (cP).

### 5.10. Histopathology

Samples from the intestine, liver and kidneys were fixed in 10% neutral-buffered formalin for 48–72 h and embedded in paraffin by a routine procedure. Dewaxed 3–5 μm thick sections were stained with haematoxylin and eosin (H–E).

### 5.11. Microbiological Analysis

During the evisceration process, one cecum from each bird was aseptically removed and forwarded for further bacteriological analysis within 24 h. The content of each cecum was weighted and co-placed by a tenfold quantity of Maximum Recovery Diluent (MRD, Oxoid), in a sterile plastic Stomacher bag. Following homogenization for 3 min in a Stomacher bag, 1 mL of homogenate was further diluted, by the 10fold dilution method. Finally, 1ml of each dilution was suspended in a sterile petri dish, in duplicate and covered by the appropriate medium, cooled at 45 °C.

For the cultivation of *Escherichia coli*, the ChromoBio^®^ TBX agar (Biolab, Budapest, Hungary) was used, and all green colonies were countered according to the ISO16649-2: 2001 after incubation for 24 h, at 44 °C, under aerobic conditions. Moreover, Lactobacilli were measured at the genus level, according to the ISO 15214:1998 after aerobic incubation for 72 h at 37 °C on de Man, Rogosa and Sharpe agar (MRS, Biolab). Tryptose Sulfite Cycloserine (TSC) agar was used for the enumeration of *Clostridium* spp. according to the ISO7937:2004. Finally, Bifidus Selective Medium (BSM, Sigma), was used for the enumeration of *Bifidobacteria* according to the ISO29981:2010. For *Clostridium* spp. and *Bifidobacteria*, plates were incubated anaerobically for 48 and 72 h, respectively. The anaerobic environment was generated using Oxoid™ AnaeroGen™ 2.5 L Sachet and was confirmed using Anaerotest^®^ (Merck 1.15112). Results were expressed as base-10 logarithm colony-forming units per gram of caecal digesta.

### 5.12. Statistical Analysis

The statistical analysis was conducted using the statistical software SPSS^®^ Statistics Ver. 25 (IBM Corp, Armonk, NY, USA) and the statistical language R [67]. Univariate descriptive statistics were evaluated for all examined parameters and results were expressed as mean ± standard deviation. The Kolmogorov–Smirnov test was used to assess the normality assumption for all quantitative variables. One-way Analysis of Variance (ANOVA) was performed in order to examine the main effect of treatment on the population mean values of parameters measured once followed by a post-hoc analysis through Tukey’s or Duncan’s tests after examining the homogeneity of variance assumption.

The effect of treatment on variables violating the normality assumption was assessed by the non-parametric Kruskal–Wallis test followed by pair-wise comparisons through the Mann–Whitney test using Bonferroni’s correction. In order to examine the effect of treatment on continuous parameters measured at different timestamps, Linear Mixed Effects (LME) modelling was used [68]. All statistical analyses were conducted using the statistical language R [67] (and the function lmer from package lme4). The *p*-values for the fixed component of the model were calculated from a F-test based on the Kenward–Roger approach [69] in order to get approximate degrees of freedom. In all tests, a difference was considered statistically significant when the *p*-value (significance) was less than 0.05 (*p* ≤ 0.05). All the tests conducted were two-tailed (non-directional) in the sense that the alternative hypothesis is that the measures tested are not equal.

## Figures and Tables

**Table 1:** The effect of mycotoxins Aflatoxin B1 (AFB1) and Ochratoxin A (OTA) and the multicomponent mycotoxin detoxifying agent (MMDA) on the performance in broiler chicks ($\bar{X}$ ± Std).

Parameter	Period	Group A Negative Control	Group B Mycotoxins	Group C Mycotoxins+ MMDA 1 g/kg Feed	Group D Mycotoxins+ MMDA 3 g/kg Feed
Body weight (g)	1 d	38 ± 2	39 ± 3	39 ± 3	39 ± 2
	7 d	131 ± 10 ^a^	132 ± 9 ^a^	126 ± 12 ^b^	131 ± 9 ^a^
	15 d	384 ± 40 ^a#^	370 ± 27 ^a#^	369 ± 32 ^a#^	382 ± 37 ^a#^
	25 d	1086 ± 10 ^a^	1003 ± 74 ^b^	1007 ± 96 ^b^	1023 ± 104 ^b^
	32 d	1763 ± 125 ^a^	1610 ± 112 ^b^	1622 ± 123 ^b^	1599 ± 130 ^b^
	42 d	2605 ± 217 ^a^	2440 ± 199 ^b^	2446 ± 192 ^b^	2477 ± 205 ^b^
Average daily weight gain (g) *	1–25 d	42 ± 1	39 ± 1	39 ± 1	39 ± 2
	25–42 d	89 ± 1	85 ± 1	85 ± 3	85 ± 5
	1–42 d	61 ± 1	57 ± 1	57 ± 1	58 ± 3
Feed conversion ratio (g:g)	1–25 d	1.62 ± 0.04 ^a^	1.72 ± 0.04 ^b^	1.73 ± 0.06 ^b^	1.73 ± 0.05 ^b^
	25–42 d	1.23 ± 0.08 ^a^	1.40 ± 0.04 ^b^	1.25 ± 0.02 ^a^	1.25 ± 0.05 ^a^
	1–42 d	1.41 ± 0.03 ^a^	1.50 ± 0.04 ^b#^	1.44 ± 0.02 ^ab#^	1.45 ± 0.05 ^ab#^
Adjusted feed conversion ratio (g:g) ^¶^	1–42 d	1.39 ± 0.02 ^a^	1.51 ± 0.05 ^b^	1.45 ± 0.02 ^ab^	1.45 ± 0.07 ^ab^

^#^ Means with such superscript in the same row suggest trend towards statistical significance (0.05 < *p* < 0.1). ^a,b^ Means with different superscripts in the same row differ significantly (*p* < 0.05). *p* values BW: day 7 (Treatment × Time interaction observed): Group A vs. Group C = 0.014; B vs. C = 0.006; C vs. D = 0.012; day 15 (Treatment × Time interaction observed): Group A vs. Group B = 0.070; A vs. C = 0.054; B vs. D = 0.090; C vs. D = 0.069; day 25 (Treatment × Time interaction observed): Group A vs. Group B < 0.001; A vs. C < 0.001; A vs. D = 0.001; day 32 (Treatment × Time interaction observed): Group A vs. Group B < 0.001; A vs. C < 0.001; A vs. D < 0.001.; day 42 (Treatment × Time interaction observed): Group A vs. Group B = 0.005; A vs. C = 0.008; A vs. D = 0.031. *p* values FCR: days 1–25 (Treatment × Time interaction observed): Group A vs. Group B = 0.003; A vs. C = 0.001; A vs. D = 0.002; days 25–42 (Treatment × Time interaction observed): Group A vs. Group B < 0.001; B vs. C < 0.001; B vs. D < 0.001; days 1–42 (Treatment × Time interaction observed): Group A vs. Group B = 0.009; B vs. C = 0.076; B vs. D = 0.10. * Comparison of mean ADWG values is presented without superscripts since differences were observed without a Treatment × Time interaction in the statistical evaluation. *p* values ADWG (Treatment × Time interaction not observed): Group A vs. Group B = 0.015; Group A vs. Group C = 0.019; Group A vs. Group D = 0.047. **^¶^** Adj.FCR: Adjusted to a standard weight of 2500 g, with a 0.2 correction per 100 g difference between actual slaughter weight and 2500 g. Adj.FCR 1–42: Group A vs. Group B = 0.005.

**Table 2:** The effect of mycotoxins AFB_1_ and OTA and the multicomponent mycotoxin detoxifying agent (MMDA) on the serum biochemical parameters in broiler chicks ($\bar{X}$ ± Std).

Parameters (Unit)	Group A Negative Control	Group B Mycotoxins	Group C Mycotoxins+ MMDA 1 g/kg Feed	Group D Mycotoxins+ MMDA 3 g/kg Feed
First Blood Sampling (25th day)				
Total Proteins * (g/dL)	2.48 ± 0.26	2.39 ± 0.28	2.23 ± 0.39	2.34 ± 0.15
Albumins * (g/dL)	1.14 ± 0.14	1.06 ± 0.14	1.02 ± 0.13	1.01 ± 0.06
ALP * (U/L)	3932 ± 788	4613 ± 1561	5682 ± 1764	4887 ± 1486
ALT * (U/L)	9.69 ± 1.89	9.81 ± 4.64	7.75 ± 2.05	6.19 ± 1.52
AST (U/L)	111 ± 33 ^c^	176 ± 23 ^a^	168 ± 27 ^a^	125 ± 13 ^b^
γ-GT * (U/L)	5.75 ± 1.57	5.75 ± 1.65	5.88 ± 1.36	6.63 ± 1.26
Glucose * (mg/dL)	226 ± 22	213 ± 27	219 ± 14	228 ± 9
Cholesterol (mg/dL)	139 ± 15 ^a#^	129 ± 17 ^a^	127 ± 16 ^a#^	138 ± 17 ^a#^
Second Blood Sampling (42nd day)				
Total Proteins * (g/dL)	2.96 ± 0.69	2.64 ± 0.71	2.86 ± 0.87	2.69 ± 0.26
Albumins * (g/dL)	1.40 ± 0.30	1.25 ± 0.23	1.31 ± 0.34	1.24 ± 0.19
ALP * (U/L)	1727 ± 568	2005 ± 645	1796 ± 628	2012 ± 418
ALT * (U/L)	5.19 ± 1.76	5.8 ± 1.61	5.63 ± 2	5.25 ± 1.98
AST (U/L)	126 ± 14 ^a^	230 ± 39 ^b^	216 ± 39 ^b^	226 ± 22 ^b^
γ-GT * (U/L)	7.56 ± 2.16	8.27 ± 2.4	7.75 ± 2.65	7.81 ± 2.48
Glucose * (mg/dL)	202 ± 24	208 ± 31	214 ± 29	220 ± 20
Cholesterol (mg/dL)	112 ± 18 ^ab^	113 ± 14 ^ab^	121 ± 16 ^b^	107 ± 16 ^a^

^#^ Means with symbol superscript in the same row suggest trend towards statistical significance (0.05 < *p* < 0.1). * Comparison of mean values of Albumins, glucose, ALP, ALT γ-GT are presented without superscripts since differences were observed without a Treatment × Time interaction in the statistical evaluation. *p* values Total Proteins at both blood samplings (Absence of Treatment × Time interaction observed): Group A vs. Group B = 0.096; A vs. C = 0.068. *p* values Albumins at both blood samplings (Absence of Treatment × Time interaction observed): Group A vs. Group B = 0.027; A vs. C = 0.022; A vs. D = 0.005. *p* values Glucose at both blood samplings (Absence of Treatment × Time interaction observed): Group B vs. Group D = 0.023. *p* values ALP at both blood samplings (Absence of Treatment × Time interaction observed): Group A vs. Group C = 0.082; A vs. D = 0.047. *p* values ALT at both blood samplings (Absence of Treatment × Time interaction observed): Group A vs. Group D = 0.021; B vs. D = 0.007. ^a,b,c^ Means with different superscripts in the same row differ significantly (*p* < 0.05) and Treatment × Time interaction at the specific parameter statistical evaluation was observed. *p* values Cholesterol at First blood sampling (Treatment × Time interaction observed): Group A vs. Group C = 0.072; C vs. D = 0.090; at 2nd blood sampling (Treatment × Time interaction observed): Group C vs. Group D = 0.012. *p* values AST at First blood sampling (Treatment × Time interaction observed): All comparisons among groups with different superscripts < 0.001, except Group A vs. Group D = 0.007; at 2nd blood sampling (Treatment × Time interaction observed): Group A vs. all groups < 0.001.

**Table 3:** The effect of mycotoxins AFB_1_ and OTA and the multicomponent mycotoxin detoxifying agent (MMDA) on the gross lesions (intestine, gizzard, and liver) and physicochemical measurements of intestinal content (pH and viscosity) in broiler chicks ($\bar{X}$ ± Std).

	Group A Negative Control	Group B Mycotoxins	Group C Mycotoxins+ MMDA 1 g/kg Feed	Group D Mycotoxins+ MMDA 3 g/kg Feed
	Gross Lesions score			
Intestine ^#^	1.44 ± 0.82	1.63 ± 0.71	1.65 ± 1.05	1.74 ± 0.79
Gizzard ^ǁ^	0.23 ± 0.42 ^a^	0.18 ± 0.5 ^a^	0.08 ± 0.27 ^a^	0.03 ± 0.16 ^b^
Liver ^¶^	0.1 ± 0.3	0.15 ± 0.43	0.25 ± 0.44	0.3 ± 0.46
	pH of Intestinal Digesta			
Duodenum	5.88 ± 0.53 ^a^	5.95 ± 0.55 ^b^	6.02 ± 0.19 ^b^	5.98 ± 0.11 ^b^
Jejunum	5.89 ± 0.17 ^a^	6.08 ± 0.15 ^b^	5.94 ± 0.2a ^b^	5.84 ± 0.15 ^a^
Ileum	6.39 ± 0.65 ^a^	5.84 ± 0.88 ^ab^	5.57 ± 0.65 ^b^	5.33 ± 0.59 ^b^
Caeca	6.01 ± 0.36 ^a^	5.97 ± 0.32 ^a^	6.18 ± 0.22 ^ab^	6.41 ± 0.36 ^b^
	Viscosity of Intestinal Digesta			
Jejunum	1.39 ± 0.46	1.57 ± 0.8	1.28 ± 0.39	1.5 ± 0.48
Ileum	1.47 ± 0.56	1.58 ± 0.64	1.14 ± 0.15	1.41 ± 0.46

^a,b^ Means with different superscripts in the same row differ significantly (*p* < 0.05). *p* values Gizzard lesions: Group A vs. Group D *p* = 0.042. *p* values pH duodenum: Group A vs. Group B = 0.006; A vs. C = 0.012; A vs. D < 0.001; pH Jejunum: Group A vs. Group B = 0.011; B vs. D = 0.001; pH Ileum: Group A vs. Group C = 0.01; A vs. D < 0.001; pH Caeca: Group A vs. Group D = 0.007; B vs. D = 0.002. **^#^** Enteritis gross lesions 0–3 scoring system: 0 = normal intestinal wall, 1 = hyperemia of intestinal wall and/or undigested intestinal content in the last third of intestine, 2 = congestion and thickness of intestinal wall and/or watery intestinal content; 3 = hemorrhages of intestinal wall. ^ǁ^ Gizzards gross lesions 0–2 scoring system: 0 = healthy gizzard (absence of lesions); 1 = moderately affected gizzard, if erosions in the koilin layer were present; 2 = severely affected gizzard (ulcers of the cuticula of the gizzard were noted). **^¶^** Livers gross lesions 0–2 scoring system: 0 = absence of lesions; 1 = liver congestion and/or gallbladder distension and wall thickening and/or bile discoloration; 2 = necrotic lesions in the liver.

**Table 4:** The effect of mycotoxins AFB_1_ and OTA and the multicomponent mycotoxin detoxifying agent (MMDA) on the caecal microbiota in broiler chicken ($\bar{X}$ ± Std).

	Group A Negative Control	Group B Mycotoxins	Group C Mycotoxins+ MMDA 1 g/kg Feed	Group D Mycotoxins+ MMDA 3 g/kg Feed
Caecal weight	8.36 ± 2.54	9.1 ± 3.07	10.44 ± 4.14	9.61 ± 2.72
*E. coli*	4.58 ± 0.87 ^ab^	5.44 ± 0.94 ^b^	4.04 ± 0.94 ^a^	4.17 ± 1.08 ^a^
*Clostridium* spp.	4.47 ± 0.81	4.36 ± 0.74	3.92 ± 0.82	4.19 ± 1.00
*Lactobacillus* spp.	6.36 ± 0.66	6.47 ± 0.62	6.46 ± 0.77	6.51 ± 0.66
*Bifidobacterium* spp.	5.34 ± 0.62	4.91 ± 0.95	5.29 ± 1.04	5.12 ± 0.92

^a,b^ Means with different superscripts in the same row differ significantly (*p* < 0.05). *p* values *E. coli* counts: Group B vs. C = 0.006; B vs. D = 0.012. Bacterial counts are expressed as base-10 logarithm colony-forming units per gram of caecal digesta.

## Data Availability

None of the data presented were deposited in an official repository.

## Supplementary Materials

The following are available online at https://www.mdpi.com/article/10.3390/toxins13060367/s1: Table S1: ingredients and analysis of the starter diets (1 to 25 d of age), Table S2: ingredients and calculated analysis of the grower diets (26 to 42 d of age).
