# Supplementary material for: Investigation of a Novel Multicomponent Mycotoxin Detoxifying Agent in Amelioration of Mycotoxicosis Induced by Aflatoxin-B1 and Ochratoxin A in Broiler Chicks
Source: Toxins (Basel). 2021 May 21;13(6):367. doi: 10.3390/toxins13060367 (PMC8224362; doi:10.3390/toxins13060367)
Supplement: Supplementary file 1 [file toxins-13-00367-s001.zip › toxins-supplementary conversion.pdf]

Supplementary Materials: Investigation of a Novel Multicomponent Mycotoxin Detoxifying Agent in Amelioration of Mycotoxicosis Induced by Aflatoxin-B1 and Ochratoxin A in Broiler Chicks

Vasilios Tsiouris, Panagiotis Tassis, Jog Raj, Tilemachos Mantzios, Konstantinos Kiskinis, Marko Vasiljević, Nikola Delić, Evanthia Petridou, Georgia D. Brellou, Zoe Polizopoulou, Nikolaos Mittas and Ioanna Georgopoulou

Table S1. Ingredients and analysis of the starter diets in broiler chicks (1 to 25 d of age).

Ingredients (g/kg Feed)	Treatment Groups [1]			
	Group A Negative Control	Group B Mycotoxins	Group C Mycotoxins+ MMDA 1 g/kg Feed	Group D Mycotoxins+ MMDA 3 g/kg Feed
Corn	442,5	421.5	421.5	421.5
Soybean meal (CP 47%)	275	275	275	275
Wheat	120	120	119	117
Molasses	20	20	20	20
Milk powder	30	30	30	30
Soybean oil	28	28	28	28
Gluten	25	25	25	25
Calcium	15	15	15	15
Lysine HCL	3	3	3	3
Sodium sulphate	2	2	2	2
Calcium formic	0.5	0.5	0.5	0.5
Methionine	1	1	1	1
Threonine	1.2	1.2	1.2	1.2
Oreganum oil	0.8	0.8	0.8	0.8
Fish meal	30	30	30	30
Vitamin premix [2]	2	2	2	2
Nutrients premix [3]	2	2	2	2
Salt	2	2	2	2
Test Product [4]	-	-	1	3
Corn spiked with AFB_1	-	12	12	12
Corn spiked with OTA	-	9	9	9
**Calculated analysis (g/kg)**				
Moisture	11.87	11.87	11.87	11.87
Dry matter	87.99	87.99	87.99	87.99
Crude protein	23	23	23	23
Crude fibre	2.38	2.38	2.38	2.38
Crude fat	5.55	5.55	5.55	5.55
Crude Ash	7.19	7.19	7.19	7.19
Starch	35.44	35.44	35.44	35.44
Methionine	0.45	0.45	0.45	0.45
Methionine + Cysteine	0.89	0.89	0.89	0.89
Lysine	1.32	1.32	1.32	1.32
Threonine	0.92	0.92	0.92	0.92
Tryptophan	0.24	0.24	0.24	0.24
Arginine	1.34	1.34	1.34	1.34
Valine	1.08	1.08	1.08	1.08
Leucine	1.85	1.85	1.85	1.85
Isoleucine	0.91	0.91	0.91	0.91
Linoleic acid	2.56	2.56	2.56	2.56
Calcium	1.01	1.01	1.01	1.01

Phosphorus	0.7	0.7	0.7	0.7
Sodium Chloride	0.19	0.19	0.19	0.19
Energy (Mj/kg)	12.60	12.60	12.60	12.60
**Analysed nutrient levels (g/kg)**				
Moisture	10.3	10.4	10.4	10.4
Crude ash	7.7	7.8	7.8	7.7
Crude proteins	22.6	22.1	21.9	22
Crude fat	5.6	5.5	5.4	5.5
Crude fibres	3.4	3.2	3.3	3.2
Starch	37.3	38.5	37.5	38.9
Sugars	4.2	4	3.9	4.1
Calcium	0.9	1	0.8	0.9
Phosphorus	0.7	0.6	0.7	0.6
**Mycotoxin concentration [5] (µg/kg)**				
AFB_1	<0.5	105.2	109.8	107
OTA	5.15	1055	1046	1059
DON	22.02	21.5	23.6	20.5
T-2	1.31	1.1	<1	<1
FB_1	59	55	62	54
FB_2	112	106	112	103

[1] Group A: Basal diet without mycotoxins or test product, Group B: Basal diet contaminated with 0.1 mg AFB_1/kg feed and 1 mg OTA/kg feed, Group C: Group B feed with addition of 1 g test product/kg feed, Group D: Group B feed with addition of 3 g test product/kg feed. [2] Contents per kg: 6,000,000 I.U. retinyl acetate; 2,000,000 I.U. cholecalciferol; 40,000 mg DL-α-tocopheryl acetate; 4500 mg menadione sodium bisulphite; 1500 mg thiamin; 3500 mg riboflavin; 3000 mg pyridoxine HCl; 12.5 µg cobalamin; 25,000 mg niacin; 75 mg biotin; 750 mg folic acid; 7500 mg pantothenic acid. [3] Contents per kg: 200,000 mg choline chloride; 25,000 mg Fe; 45,000 mg Zn; 65,000 mg Mn; 10,000 mg Cu; 750 mg I; 150 mg Se. [4] Multi-component mycotoxin detoxifying agent containing modified zeolite (Clinoptilolite), *Bacillus subtilis, Bacillus Licheniformis, Saccharomyces cerevisiae* cell wall and silymarin. [5] Method of detection UPLC MS/MS. Detection limits (µg/kg): AFB_1=0.5; OTA=1; FB_1, FB_2, DON=10; T-2 = 1.

Table S2. Ingredients and calculated analysis of the grower diets in broiler chicks (26 to 42 d of age).

**Ingredients (g/kg Feed)**	**Treatment Groups [1]**			
	**Group A Negative Control**	**Group B Mycotoxins**	**Group C Mycotoxins+ MMDA 1 g/kg Feed**	**Group D Mycotoxins+ MMDA 3 g/kg Feed**
Corn	437	416	416	416
Soybean meal (CP 47%)	305	305	305	305
Wheat	125	125	124	122
Molasses	16	16	16	16
Milk powder	25	25	25	25
Soybean oil	30	30	30	30
Barley	25	25	25	25
Gluten	9.2	9.2	9.2	9.2
Calcium	15.5	15.5	15.5	15.5
Lysine HCL	2	2	2	2
Sodium sulphate	2	2	2	2
Calcium formic	0.5	0.5	0.5	0.5
Methionine	1.4	1.4	1.4	1.4
Threonine	-	-	-	-
Oreganum oil	0.5	0.5	0.5	0.5
Fish meal	-	-	-	-
Vitamin premix [2]	2	2	2	2
Nutrients premix [3]	2	2	2	2
Salt	1.9	1.9	1.9	1.9
Test Product [4]	-	-	1	3
Corn spiked with AFB_1	-	12	12	12
Corn spiked with OTA	-	9	9	9
**Calculated analysis (g/kg)**				
Moisture	11.91	11.91	11.91	11.91
Dry matter	87.34	87.34	87.34	87.34
Crude protein	21.19	21.19	21.19	21.19
Crude fibre	2.46	2.46	2.46	2.46
Crude fat	5.46	5.46	5.46	5.46
Crude Ash	5.22	5.22	5.22	5.22
Starch	36.75	36.75	36.75	36.75
Methionine	0.45	0.45	0.45	0.45
Methionine + Cysteine	0.88	0.88	0.88	0.88
Lysine	1.24	1.24	1.24	1.24
Threonine	0.79	0.79	0.79	0.79
Tryptophan	0.25	0.25	0.25	0.25
Arginine	1.32	1.32	1.32	1.32
Valine	1.04	1.04	1.04	1.04
Leucine	1.72	1.72	1.72	1.72
Isoleucine	0.86	0.86	0.86	0.86
Linoleic acid	2.64	2.64	2.64	2.64
Calcium	1.00	1.00	1.00	1.00
Phosphorus	0.62	0.62	0.62	0.62
Sodium Chloride	0.22	0.22	0.22	0.22
Energy (Mj/kg)	12.54	12.54	12.54	12.54
**Analysed nutrient levels (g/kg)**				
Moisture	10.4	10.4	10.5	10.4
Crude ash	6.8	6.4	6.6	6.6
Crude proteins	20.3	20.6	20.8	20.6
Crude fat	5.7	5.7	5.7	5.5
Crude fibres	3.2	3.3	3.1	3.3
Starch	38.2	38.8	38.6	38
Sugars	3.9	4.1	4	3.9
Calcium	0.9	0.9	1	0.9
Phosphorus	0.6	0.6	0.6	0.6
**Mycotoxin concentration [5] (μg/kg)**				

AFB_1	1.07	116	111.4	114.6
OTA	<1	1068	1060	1055
DON	16.9	15.7	14.4	13.1
T-2	1.01	<1	<1	<1
FB_1	62	66	53	57
FB_2	142	138	133	130

[1] Group A: Basal diet without mycotoxins or test product, Group B: Basal diet contaminated with 0.1 mg AFB_1/kg feed and 1 mg OTA/kg feed, Group C: Group B feed with addition of 1 g test product/kg feed, Group D: Group B feed with addition of 3 g test product/kg feed. [2] Contents per kg: 5,000,000 I.U. retinyl acetate; 1,500,000 I.U. cholecalciferol; 25,000 mg DL-α-tocopheryl acetate; 3500 mg menadione sodium bisulphite; 1500 mg thiamin; 3000 mg riboflavin; 3000 mg pyridoxine HCl; 12.5 μg cobalamin; 20,000 mg niacin; 75 mg biotin; 600 mg folic acid; 6000 mg pantothenic acid. [3] Contents per kg: 200,000 mg choline chloride; 25,000 mg Fe; 45,000 mg Zn; 65,000 mg Mn; 10,000 mg Cu; 750 mg I; 150 mg Se. [4] Multi-component mycotoxin detoxifying agent containing modified zeolite (Clinoptilolite), *Bacillus subtilis*, *Bacillus Licheniformis*, *Saccharomyces cerevisiae* cell wall and silymarin. [5] Method of detection UPLC MS/MS. Detection limits (μg/kg): AFB_1=0.5; OTA=1; FB_1, FB_2, DON=10; T-2 = 1.
